# European COMPARative Effectiveness research on blended Depression treatment versus treatment-as-usual (E-COMPARED): study protocol for a randomized controlled, non-inferiority trial in eight European countries

**DOI:** 10.1186/s13063-016-1511-1

**Published:** 2016-08-03

**Authors:** Annet Kleiboer, Jan Smit, Judith Bosmans, Jeroen Ruwaard, Gerhard Andersson, Naira Topooco, Thomas Berger, Tobias Krieger, Cristina Botella, Rosa Baños, Karine Chevreul, Ricardo Araya, Arlinda Cerga-Pashoja, Roman Cieślak, Anna Rogala, Christiaan Vis, Stasja Draisma, Anneke van Schaik, Lise Kemmeren, David Ebert, Matthias Berking, Burkhardt Funk, Pim Cuijpers, Heleen Riper

**Affiliations:** 1 Section Clinical Psychology, Vrije Universiteit Amsterdam and EMGO+ Institute for Health Care and Research, Van der Boechorststraat 1, 1081 BT Amsterdam, The Netherlands; 2 Department of Psychiatry, VU University Medical Centre and EMGO+ Institute for Health Care and Research, Amsterdam, The Netherlands; 3 Department of Health Sciences, Vrije Universiteit Amsterdam and EMGO+ Institute for Health and Care Research, Amsterdam, The Netherlands; 4 Department of Behavioural Sciences and Learning, Swedish Institute for Disability Research, Linköping University, Linköping, Sweden; 5 Department of Clinical Neuroscience, Psychiatry Section, Karolinska Institutet, Stockholm, Sweden; 6 Department of Clinical Psychology and Psychotherapy, University of Bern, Bern, Switzerland; 7 Department of Psychology and Technology, Jaume University, Castellon, Spain; 8 Department of Personalidad, Evaluación y Tratamiento Psicológicos, Valencia, Spain; 9 URC-ECO, Ile-de-France (AP-HP), Paris, France; 10 Department of Population Health, London School of Hygiene and Tropical Medicine, London, UK; 11 Department of Psychology, Szkoła Wyzsza Psychologii Społeczne, University of Social Sciences and Humanities, Warsaw, Poland; 12 Department of Clinical Psychology, Philipps University, Marburg, Germany; 13 Institut für elektronische Geschäftsprozesse, Leuphana University Lüneburg, Lüneburg, Germany

**Keywords:** Internet-based treatment, Depression, Cognitive behavioral treatment (CBT), Blended treatment, Comparative effectiveness research (CER), Cost-effectiveness, Randomized controlled trial (RCT), Adults, iCBT, eHealth

## Abstract

**Background:**

Effective, accessible, and affordable depression treatment is of high importance considering the large personal and economic burden of depression. Internet-based treatment is considered a promising clinical and cost-effective alternative to current routine depression treatment strategies such as face-to-face psychotherapy. However, it is not clear whether research findings translate to routine clinical practice such as primary or specialized mental health care. The E-COMPARED project aims to gain knowledge on the clinical and cost-effectiveness of blended depression treatment compared to treatment-as-usual in routine care.

**Methods/design:**

E-COMPARED will employ a pragmatic, multinational, randomized controlled, non-inferiority trial in eight European countries. Adults diagnosed with major depressive disorder (MDD) will be recruited in primary care (Germany, Poland, Spain, Sweden, and the United Kingdom) or specialized mental health care (France, The Netherlands, and Switzerland). Regular care for depression is compared to “blended” service delivery combining mobile and Internet technologies with face-to-face treatment in one treatment protocol. Participants will be followed up at 3, 6, and 12 months after baseline to determine clinical improvements in symptoms of depression (primary outcome: Patient Health Questionnaire-9), remission of depression, and cost-effectiveness. Main analyses will be conducted on the pooled data from the eight countries (*n* = 1200 in total, 150 participants in each country).

**Discussion:**

The E-COMPARED project will provide mental health care stakeholders with evidence-based information and recommendations on the clinical and cost-effectiveness of blended depression treatment.

**Trial registration:**

France: ClinicalTrials.gov NCT02542891. Registered on 4 September 2015; Germany: German Clinical Trials Register DRKS00006866. Registered on 2 December 2014; The Netherlands: Netherlands Trials Register NTR4962. Registered on 5 January 2015; Poland: ClinicalTrials.Gov NCT02389660. Registered on 18 February 2015; Spain: ClinicalTrials.gov NCT02361684. Registered on 8 January 2015; Sweden: ClinicalTrials.gov NCT02449447. Registered on 30 March 2015; Switzerland: ClinicalTrials.gov NCT02410616. Registered on 2 April 2015; United Kingdom: ISRCTN registry, ISRCTN12388725. Registered on 20 March 2015.

**Electronic supplementary material:**

The online version of this article (doi:10.1186/s13063-016-1511-1) contains supplementary material, which is available to authorized users.

## Background

Depression is a common mental disorder with a negative impact on mental well-being, quality of life, and social and work-related functioning both in the short and longer term [1]. Additionally, depression is associated with increased morbidity, mortality, health care utilization, and health care costs [2–4]. On a population level, depression is one of the most costly diseases. In the European Union, 30 million citizens are affected by depression and the economic costs of depression were estimated at €92 billion in 2010 and are still rising [5, 6]. The majority of these costs is caused by lost productivity [5]. In combination with the fact that depression is highly prevalent among the working-age population [7], depression poses a significant societal and economic burden to European society.

Depression can be treated effectively with pharmacological treatment, psychotherapy, especially cognitive-behavioral therapy (CBT), or a combination of both [8–12]. Antidepressants are widely prescribed, both in primary and specialized mental health care settings, while non-pharmacologic treatments, such as psychotherapeutic interventions, are offered to a lesser degree in most of Europe, and are virtually absent in primary care in some countries such as Portugal [13]. Moreover, despite the availability of effective treatments, the proportion of adults with depression who actually seek or receive treatment is limited with estimates ranging between 35 and 45 % in higher-income countries [14, 15] indicating that the treatment of depression leaves ample room for improvement. Thus, European health care systems face the challenge of improving access to cost-effective treatments to relieve the burden of depression [16].

Internet-based depression treatment is considered a promising clinical and cost-effective alternative to current routine depression treatment strategies such as face-to-face psychotherapy. Ample research has demonstrated the clinical effectiveness of Internet-based treatment for depression when delivered with minimal guidance in controlled research settings [17] with effect sizes that are comparable to face-to-face interventions [18]. It is also unclear whether Internet-based depression treatments are cost-effective, which is considered an important next step in research [19]. In addition, it is not yet clear to what extent these findings translate to routine clinical practice such as primary care or specialized mental health care. The patient populations in these settings are more heterogeneous than those in controlled research settings and the delivery of services in these settings is more complex.

Blended depression treatment is a relatively new treatment format where online and face-to-face interventions are integrated into one treatment protocol [20, 21]. Blended depression treatment formats are of particular interest to primary and specialized treatment centers for several reasons [22, 23]. Patients often present with complex clinical problems and may need more intensive guidance than Internet-based treatments alone are able to offer [24]. Blended treatment delivery would allow therapists to focus on process-related treatment components during the face-to-face sessions, such as patient-specific needs, discussion of thoughts and feelings, and treatment evaluation, while more practical therapy aspects can be delivered in the online sessions (e.g., psychoeducation, homework, and symptom monitoring) [25, 26]. In addition, treatment in specialized mental health care settings is costly. Both in primary and specialized mental health care, patients often return for follow-up consultations which provides an opportunity to introduce blended treatments. It is expected that implementation of blended Internet-based treatments may reduce the number of face-to-face sessions needed while mental health gains remain the same or even increase. Thus, there are a number of advantages associated with blended depression treatment which may improve the efficiency of the health care system on the whole if these are implemented on a wider scale.

Comparative effectiveness research (CER) is designed to inform health care decisions with the aim of improving patient outcomes, quality of life, and cost-containment. The overall objective of E-COMPARED is to provide mental health care stakeholders, including patients, health care professionals, health insurers, mental health service providers, and policy-makers, with evidence-based information and recommendations about the clinical effectiveness and cost-effectiveness of blended depression treatment in comparison with treatment-as-usual (TAU) in Europe. The study will be conducted in eight European countries with different diversities and geographical spread including countries that are frontrunners in the field of Internet-based treatments (The Netherlands, Sweden, and the United Kingdom (UK)), countries in which the field has been evolving rapidly (Germany, Spain, and Switzerland), and countries with very little expertise and experience in this area (France and Poland). Thus, the outcome of the project will take into account these different situations and the results will inform the routine practice of specific mental health service organizations in Europe. The aim of this paper is to describe the protocol of the multinational study that evaluates the effectiveness and cost-effectiveness of blended cognitive behavioral therapy (bCBT) for adults with major depressive disorder (MDD) in comparison with TAU in Europe both in primary and specialized mental health care settings. It is hypothesized that bCBT is clinically non-inferior (i.e., not less effective) as compared to TAU, but that it is cost-effective since we expect that less therapist time is needed to deliver treatment resulting in lower treatment costs.

## Method/design

### Design

This is a two-arm, randomized controlled, non-inferiority trial, with an economic evaluation alongside, in eight countries in Europe (France, Germany, The Netherlands, Poland, Spain, Sweden, Switzerland, and the UK). The non-inferiority design was chosen to test whether bCBT is not less effective than TAU [27]. The trials will be conducted in routine primary care (sites: Germany, Poland, Spain, Sweden, and the UK) or specialized mental health care (sites: France, The Netherlands, and Switzerland). Respondents in both conditions will be followed until 12 months after baseline (measures will be taken at baseline, 3 months, 6 months, and 12 months).

### Participants

**Fig. 1:**
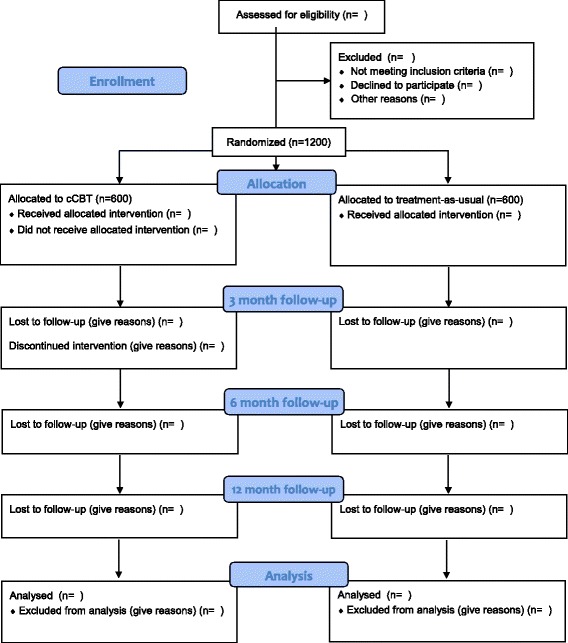
Flow of participants through the trial

Recruitment procedures are country- and setting-specific (see Table 1). See Fig. 1 for the flow of patients through the study and attachments (Additional files 1 and 2) for an overview of study procedures and a checklist in line with Standard Protocol Items: Recommendations for Interventional Trials (SPIRIT) [28]. In all countries, the following inclusion and exclusion criteria will be applied uniformly.

**Table 1:** Recruitment procedure and treatment setting per country

Country	Treatment setting	Recruitment procedure
FR	Specialized mental health care	New or regular patients recruited through cognitive behavioral therapy (CBT) therapists from 11 expert centers throughout France
DE	Primary care	Recruitment in the waiting room of general practices
NL	Specialized mental health care	New patients recruited through mood disorder departments of three outpatient clinics in Amsterdam and Leiden
PL	Primary care	Recruitment through primary mental health care centers and therapists trained at postgraduate CBT programs conducted in five major cities (Warsaw, Sopot, Poznan, Katowice, and Wroclaw)
ESP	Primary care	Recruitment through several primary care centers of patients who report to the GP with depression
SE	Primary care	Recruitment through routine primary care clinics in Stockholm and Linköping
CH	Specialized mental health care	Recruitment through two outpatient clinics (Bern and Zurich) and individual therapists
UK	Primary care	Recruitment through the NHS program “Improving Access to Psychological Therapies (IAPT)” in the London region. IAPT is a service to increase primary mental health care offering low-intensity treatment to GP-registered adults

Inclusion criteria: (1) being 18 years of age or older, (2) meeting *Diagnostic and Statistical Manual of Mental Disorders* (DSM-IV) diagnostic criteria for MDD as confirmed by the telephone-administered MINI International Neuropsychiatric Interview (M.I.N.I) version 5.0 [29], and (3) minimal to severe symptoms of depression based on a score of 5 or higher on the Patient Health Questionnaire-9 (PHQ-9) screening questionnaire [30].

Exclusion criteria: (1) current high risk for suicide according to the M.I.N.I. interview section C, (2) psychiatric comorbidity: substance dependence, bipolar affective disorder, psychotic illness, obsessive compulsive disorder, as established during the M.I.N.I. interview, (3) currently receiving psychological treatment for depression in primary or specialized mental health care, (4) being unable to comprehend the spoken and written language of the country where the study is conducted (i.e., Dutch in The Netherlands, German in Germany, English in the UK, Polish in Poland, Swedish in Sweden, German in Switzerland, French in France, and Spanish in Spain), (5) not having access to a computer with fast Internet connection (i.e., broadband or comparable), and (6) not having a smartphone that is compatible with the mobile component of the intervention that is offered, or not willing to carry a smartphone if one is provided with one by the research team for the study duration.

### Sample size calculation

The sample size calculation is based on the non-inferiority design and calculated for the primary clinical outcome, symptoms of depression at 3 months after baseline. The non-inferiority margin was set at Cohen’s *d* = 0.20 which is a conservative estimate of the subjective minimal important difference that is noticeable by depressed patients [31]. To test the hypothesis that bCBT is not inferior to TAU, a total of 1052 patients is required. With this number of patients we are 90 % certain (power of .90) that the lower limit of the two-sided 95 % confidence interval will truly be above the non-inferiority limit Cohen’s *d* = −0.20. To allow for expected study dropout and variation between settings the total number of participants to be recruited will be 1200, 150 patients in each country.

### Randomization

Randomization will be conducted centrally by the (PI) organization (VU Amsterdam) by a team of independent researchers (the randomization team). The randomization team allocates patients to treatment in all countries except for France where the allocation process will be automated and the UK where the allocation process will be outsourced to a local independent researcher as for logistical reasons the randomization outcome should be instantly available. Randomization will take place at an individual level, stratified by country, after the eligibility and baseline assessment. Further stratification on recruitment location will be applied in France (11 centers), Switzerland (3 centers), The Netherlands (3 centers), and Sweden (two cities). The randomization team will create the allocation scheme with a computerized random number generator (Random Allocation Software) at an allocation ratio of 1:1. Block randomization will be used with variable block sizes that vary between 8 and 14 allocations per block (in France block sizes of 2 and 4 will be applied because of the large number of strata). Subjects will be randomized into two groups: bCBT or TAU. All investigators and clinicians will be unknown to the randomization scheme. Blinding for treatment allocation is not possible as it will be clear to both therapists and patients whether the treatment is blended or not. However, outcome assessors will be blinded.

### Assessment measures

An overview of the measures and the time of assessment is provided in Table 2. All questionnaire measures are completed online.

**Table 2:** Overview of measures

Variable	Instrument	Screening Baseline	3 months	6 months	12 months
Questions taken from patients					
Demographics		X			
Current treatment		X			
Diagnostic interview	M.I.N.I.	X			X
Depressive symptoms	PHQ-9 and QIDS-SR16	X	X	X	X
Quality of life	EQ-5D-5L	X	X	X	X
Societal costs	TIC-P	X	X	X	X
Treatment preference		X			
Patient expectancy	CEQ	X			
Working alliance	WAI-SF		X^c^		
Technology alliance	TAI-OT^a^		X^c^		
Client satisfaction	CSQ		X^c^		
Satisfaction with the online program	SUS^a^		X^c^		
Questions taken from therapists					
Working alliance	WAI-SF		X^c^		
Satisfaction with the online program	SUS^a,b^		X^c^		

^a^Offered to condition receiving blended treatment only

^b^Has to be completed once per therapist after completion of the first treatment

^c^Questionnaires may be taken at 6 months when the duration of treatment is longer than 3 months

*CEQ* Credibility and Expectancy Questionnaire, *CSQ* Client Satisfaction Questionnaire-8, *EQ-5D-5L* EuroQol 5 dimensions 5 levels, *M.I.N.I.* MINI International Neuropsychiatric Interview, *PHQ-9* Patient Health Questionnaire-9, *QIDS-SR16* Quick Inventory of Depressive Symptomatology, *SUS* System Usability Scale, *TAI-OT* Technology Alliance Inventory-Online Therapy, *TIC-P* Trimbos and iMTA Questionnaires on Costs Associated with Psychiatric Illness, *WAI-SF* Work Alliance Inventory short form

#### Primary outcome

The primary outcome measure is symptoms of depression as assessed with the Patient Health Questionnaire-9 (PHQ-9) at 3 months [30]. The PHQ-9 is a multipurpose instrument that was developed for use in primary care but has been validated in different patient populations such as primary care, the general population, specialized mental health care, and patients with somatic disease [32, 33]. The questionnaire is used frequently in clinical trials to assess the outcomes of treatment [34]. The nine items are each scored on a 0–3 scale with the total score ranging from 0–27 and higher scores indicating more severe depression. The PHQ-9 has been shown to have good psychometric properties [32, 33].

#### Secondary outcomes

The 16-item self-report version of the Quick Inventory of Depressive Symptomatology Self-Report (QIDS-SR) [35] will be used in addition to the PHQ-9 to measure symptoms of depression. The patient QIDS-SR consists of 16 items (each item scores 0–3) and includes symptom domains of MDD based on the DSM-IV. The QIDS-SR has shown good psychometric properties in several clinical populations [34].

*A diagnosis of depression* will be established with the M.I.N.I. International Neuropsychiatric Interview (M.I.N.I.) version 5.0. The M.I.N.I. is a structured diagnostic interview based on the DSM-IV and the *International Classification of Diseases* (ICD-10) criteria. The M.I.N.I. has been translated to 65 languages and is used for both clinical and research practice. The interview compares well with the Structural Clinical Interview for DSM-IV Disorders (SCID) [29] and the Composite International Diagnostic Interview (CIDI) [29, 36]. The full M.I.N.I. 5.0, with exception of sections M (Anorexia nervosa), N (Bulimia nervosa), and P (Antisocial personality disorder), will be administered at baseline to assess lifetime and current depression, and current comorbid disorders that often co-occur with and predict the onset of depression (anxiety disorders and post-traumatic stress disorder; PTSD), and other comorbid disorders that are an exclusion criteria in this study (i.e., substance dependence, bipolar affective disorder, psychotic illness, and obsessive compulsive disorder). At the 12-month follow-up, the depression, anxiety and PTSD sections will be administered again to assess recovery of depression and status of the frequently co-occurring disorders of anxiety and PTSD.

*Quality of life* will be assessed with the EQ-5D-5L [37]. The EQ-5D-5L is a self-report questionnaire which measures health-related quality of life and enables conversion to utility scores to calculate quality-adjusted life years (QALYs). The EQ-5D-5L consists of five dimensions: mobility, self-care, ordinary activities, discomfort, and mood state related to anxiety or depression. For each dimension there are five severity levels defined ranging from no problems to many problems [38]. The EQ-5D-5L has been translated into more than 100 different language versions. The EQ-5D-5L health states will be converted to utility scores using country-specific preference weights if available. Otherwise, the UK preference weights will be used. QALYs will be calculated by multiplying the utilities with the amount of time a patient has spent in a particular health state. Transitions between health states will be linearly interpolated.

#### Cost measures

Costs will be measured from a societal perspective. Health service uptake, use of informal care, and lost productivity due to illness will be measured with an adapted version of the Trimbos and iMTA Questionnaires on Costs Associated with Psychiatric Illness (TiC-P) [39]. The TiC-P is a self-report questionnaire and consists of two different parts that can be administered separately. Part I will be used to assess the participants’ health care utilization and medication use. Part II (short form Health and Labor Questionnaire; SF-HLQ) measures lost productivity costs resulting from absenteeism (being absent from work because of illness) and presenteeism (being present at work while ill which may lead to reduced efficiency), and consists of 11 items. Health care utilization, use of informal care, and productivity losses will be valued using country-specific opportunity costs.

#### Other assessments

Several demographic variables, history of treatment for mental health problems, and treatment preferences will be measured at baseline.

The *therapeutic alliance* between therapists and patients will be assessed with the short version of the Working Alliance Inventory short form (WAI-SF). The WAI-SF is a 12-item self-report questionnaire with responses on a 5-point Likert scale ranging from 1 (never) to 5 (always) [40]. The questionnaire covers three dimensions of working alliance: (1) therapeutic goals, (2) tasks, and (3) bond, and the subscales have been shown to have good internal consistencies. Both the patient and the 10-item therapist version of the questionnaire will be administered at 3-month follow-up. The alliance between the patient and technologies will be assessed with the WAI Online Therapy questionnaire developed by Labpsitec (http://www.labpsitec.uji.es/esp/index.php) at 3-month follow-up.

*Patients’ expectancy of treatment* will be assessed with the credibility and expectancy questionnaire (CEQ) of Devilly and Borkovec [41] at baseline. Both factors (credibility and expectancy) have been shown to be stable across different populations with high internal consistency within each factor. The scale consists of six questions, with answer options rated on a 10-point scale and on a 1–100 % scale.

*Patient’s satisfaction with the treatment* will be assessed with the Client Satisfaction Questionnaire-8 (CSQ-8) [42]. This questionnaire has been translated into multiple languages and is used to measure global patient satisfaction. The questionnaire consists of eight items that are measured on a 4-point scale with total scores ranging from 8 to 32 and has shown good psychometric properties.

*Satisfaction with the platform* will be evaluated with the System Usability Scale (SUS) [43]. The SUS is a 10-item questionnaire giving a global view of subjective assessments of usability of a technology system. All items are measured on a 5-point scale ranging from strongly disagree to strongly agree. Total SUS scores have a range from 0–100. The questionnaire was found to be reliable and robust [44].

### Treatment

#### bCBT

The blended depression treatment in this study combines individual face-to-face CBT with CBT delivered through an Internet-based treatment platform with mobile phone components (either integrated in the treatment platform or as a separate system). The core components of the bCBT treatment are: (1) psychoeducation, (2) cognitive restructuring, (3) behavioral activation, and (4) relapse prevention. These will be delivered over 11–20 sessions (see Table 3). Traditional CBT treatment consists mostly of face-to-face sessions. In our bCBT treatment, the number of face-to-face sessions is reduced and replaced by online treatment modules. In this study, the ratio between the number of face-to-face sessions and the number of online modules may vary according to practice in participating countries, but a minimum of one third of the sessions should be face-to-face and a minimum of one third of the sessions should be provided online. Thus, for example, when the number of face-to-face sessions is 6, the number of online sessions lies between 3 and 12. bCBT may also include additional components such as mindfulness, coping skills training or problem solving, but these additional components cannot take up more than a quarter of the total treatment (no more than about 25 % of the face-to-face and online sessions combined). This is to prevent too much heterogeneity in the treatment programs. bCBT will be provided by CBT therapists who will receive training on how to deliver the treatment. CBT therapists will either be licensed CBT therapists, CBT therapists in training who work under supervision of an experienced licensed CBT therapist in specialized mental health care, or licensed psychologists, or psychologists in training who work under supervision of a licensed psychologist with a CBT orientation in primary care.

**Table 3:** Overview of blended treatment applied in each country

Country	Platform	Duration	Online/face-to-face	Sequencing
FR^a^	Moodbuster	16 wks	8/8	Alternate
DE^a^	Moodbuster	10–13 wks	10/5	Alternate
NL^a^	Moodbuster	20 wks	10/10	Alternate
PL^a^	Moodbuster	6–10 wks	6/7	Alternate
ESP^b^	Smiling is fun	10 wks	8/3	1-4-1-4-1
SE	Iterapi	12 wks	8/4	Alternate
CH^c^	Deprexis	18 wks	9/9	Alternate
UK^a^	Moodbuster	11 wks	5/6	Alternate

^a^Additional module on physical exercise, and problem solving

^b^Additional module on coping skills

^c^Additional modules on mindfulness, interpersonal skills, positive psychology, emotion-focused therapy, and childhood experiences

#### Internet platforms applied in this study

Various platforms are used in this study, tailored to the needs of local sites (see Table 3). All Internet platforms include: (1) a web-based interface providing the patients’ access to online CBT, (2) a mobile phone component which enables daily monitoring of mood state, cognitions, activities, social interaction, and sleep (Ecological Momentary Assessment; EMA). The mobile measures will be time and date stamped.

#### Treatment fidelity

To ensure treatment fidelity: (1) a detailed bCBT treatment manual is available at each site to guide therapists through the treatment, (2) regular contacts are organized between the therapists and the research team to prevent drift of the treatment protocols within each country, and (3) therapists register the number of sessions, the frequency of the sessions, the main strategies used in each session, the duration of each contact, and whether they have referred the patient to the online part of the treatment. Therapist activities on the platform will be assessed through track and change functionalities to measure, for example, the number of logins and type of activity in each country (log files). In some countries, therapists deliver both treatments (bCBT and TAU), because there are not sufficient therapists available in the partaking country (i.e., France) or the intake procedure does not allow a change of therapist after randomization (i.e., The Netherlands, and Switzerland). Thus, in these countries the therapist conducting the intake will also be the therapist providing the treatment. Contamination between blended depression treatment and TAU is not an insurmountable problem in this study as the contents of the face-to-face treatments may be similar and the participants in the TAU condition do not have access to the Internet-based treatment. Patients are allowed to receive pharmacotherapy in addition to the bCBT if deemed necessary by the health care professional since this reflects routine clinical practice in both treatment conditions.

#### TAU

TAU is defined as the routine care that subjects receive when they are diagnosed with depression in the specific treatment setting where they are recruited. In practice, this means that TAU may vary between countries, treatment setting, and among patients (see Table 1) and is likely to include pharmacologic treatment, psychotherapy or a combination of both. We will not interfere with TAU but we will monitor carefully which health care services are utilized by TAU patients using patient records and through self-report (including TIC-P measurements).

### Statistical approach

Data from the eight trials will be combined and multiple imputation will be used to deal with missing data. Intention-to-treat analyses (ITT) increase the risk of type I errors in non-inferiority trials and per-protocol analyses are preferred over ITT analyses [27]. Therefore, the main analysis will consist of a per-protocol analysis meaning that only those patients who have completed the treatment will be included in the analyses. ITT analyses will be used in sensitivity analyses to increase confidence in the results obtained by including all participants in the analyses independent of whether they completed the treatment or not. Multilevel regression analyses will be applied for both types of analyses, taking into account the differences between countries. Time of assessment will be treated as a fixed variable to examine the difference between groups on each occasion of measurement. Regarding the primary outcome, symptoms of depression, bCBT is considered non-inferior as compared with TAU when the two-sided 95 % confidence interval (the range of plausible differences between the two treatments) lies entirely above the standard mean difference of −0.20 which is the non-inferiority margin and the smallest clinically acceptable difference. Cohen’s *d* will be calculated to determine the magnitude of the treatment effects for continuous outcome measures, both within groups for each timepoint compared to baseline, and between groups. Effect sizes under <0.2 are deemed to be small, between 0.2 and 0.5 are deemed to be moderate, and >0.8 are deemed to be large. For dichotomous outcome measures, we will calculate the relative risk ratio.

### Economic evaluation

The economic evaluation will be first conducted on a time horizon corresponding to the trial horizon. In a second, time modeling cost-effectiveness on a longer period (a 5-year horizon) will be considered. The analysis will be performed both from the societal perspective and a health care cost perspective. Multiple imputation will be used to impute missing cost and effect data. Bivariate regression models will be used to estimate cost and effect differences while adjusting for potential confounders [45]. Incremental cost-effectiveness ratios (ICERs) will be calculated by dividing the mean difference in costs between bCBT and TAU by the difference in effects. To account for the typically skewed distribution of costs, bias-corrected and accelerated bootstrapping (5000 replications) will be used to estimate the 95 % confidence intervals around the mean cost differences and the uncertainty surrounding the ICERs. The bootstrapped ICERs will be graphically presented in cost-effectiveness planes [46]. Cost-effectiveness acceptability curves [47] will be estimated to show the probability that bCBT is cost-effective in comparison with TAU for a range of different ceiling ratios, thereby showing decision uncertainty.

### Ethical issues

Ethical approval for the trials has been obtained locally in each country. All participants provide written informed consent before taking part in the trial and are asked for their permission to share their (anonymized) data across participating E-COMPARED partners and to report on the results in publications. When data is shared among the collaborating sites and countries it will be encrypted and it will not contain identifiable information. The studies will be conducted in line with the declaration of Helsinki [48] and each trial is registered in a local clinical trial register. All researchers will follow the guidelines for Good Clinical Practice [49] and the trial outcomes will be reported in line with the Consolidated Standards of Reporting Trials (CONSORT) guidelines [50].

## Discussion

European health care systems face the challenge of improving access to cost-effective treatments to relieve the burden of depression. Comparative effectiveness research (CER) may be an appropriate method to provide evidence for improved informed decision on depression treatment with the aim to increase patient outcomes, quality of life, and cost-containment. The E-COMPARED project aims to examine the clinical and cost-effectiveness of bCBT for adults with depression compared to TAU in eight countries across Europe. It is expected that bCBT is clinically non-inferior compared to TAU, but that it is cost-effective as less therapist time is needed to deliver treatment.

This is one of the first trials to examine the effectiveness of bCBT in routine primary and specialized mental health care throughout Europe. So far, most studies have been conducted in controlled research settings in individual countries, and it is not clear how these findings translate to routine “real-world” practice or how they differ between health care systems. The E-COMPARED study is a large, multinational, randomized controlled trial with sufficient statistical power to take into account the heterogeneity between countries by pooling data. Comparison of bCBT with TAU will inform how blended depression treatment is related to current routine practice in different settings and under different circumstances.

Research in routine practice is difficult as research procedures are often constrained by the complexity of the service delivery centers and the patient population. Therefore, pragmatic decisions (i.e., external validity) have to be balanced against stringent research design requirements (i.e., internal validity). We expect that research will be even more complicated in countries that do not have much experience with Internet-based treatment or psychotherapy for depression, such as Poland and France, as we do not know to what extent this type of treatment is acceptable to patients and therapists in those countries. From a research design perspective, the differences in TAU between countries are challenging. In some countries, TAU may be primarily pharmacotherapy whereas in other countries this may be CBT or combined treatment. This is a pragmatic study, however, and where possible we have standardized study procedures, inclusion and exclusion criteria, outcome measures, and intervention components while not restricting the content of TAU in any way. The results will provide evidence-based recommendations for national and European mental health policy and decision-makers allowing them to make informed decisions regarding the dissemination and implementation of Internet-based treatments in primary and specialized care, while taking into account the different health care systems.

### Trial status

The study commenced recruitment in February 2015 and is currently recruiting.

## Abbreviations

bCBT, blended cognitive behavioral therapy; CBT, cognitive behavioral therapy; CEQ, Credibility and Expectancy Questionnaire; CER, comparative effectiveness research; CIDI, Composite International Diagnostic Interview; CSQ-8, Client Satisfaction Questionnaire-8; DSM-IV, *Diagnostic and Statistical Manual of Mental disorders*; EMA, Ecological Momentary Assessment; EQ-5D-5L, EuroQol 5 dimensions 5 levels; ICD-10, *International Classification of Diseases-10*; ICER, incremental cost-effectiveness ratio; ITT, intention-to-treat; M.I.N.I, MINI International Neuropsychiatric Interview; MDD, major depressive disorder; PHQ-9, Patient Health Questionnaire-9; PTSD, post-traumatic stress disorder; QALY, quality-adjusted life years; QIDS, Quick Inventory of Depressive Symptomatology; RCT, randomized controlled trial; SCID, Structural Clinical Interview for DSM-IV; SF-HLQ, short form Health and Labor Questionnaire; SUS, System Usability Scale; TAU, treatment as usual; TIC-P, Trimbos and iMTA Questionnaires on Costs Associated with Psychiatric Illness; WAI-SF, Work Alliance Inventory short form

## Additional files

Additional file 1: Figure 2. Schedule of enrolment, interventions, and assessments in line with SPIRIT. (DOC 58 kb) Additional file 2: SPIRIT 2013 Checklist: Recommended items to address in a clinical trial protocol and related documents. (DOC 121 kb)
