# Supplementary material for: European COMPARative Effectiveness research on blended Depression treatment versus treatment-as-usual (E-COMPARED): study protocol for a randomized controlled, non-inferiority trial in eight European countries
Source: Trials. 2016 Aug 3;17:387. doi: 10.1186/s13063-016-1511-1 (PMC4972947; doi:10.1186/s13063-016-1511-1)
Supplement: Additional file 1: Figure 2. — Schedule of enrolment, interventions, and assessments in line with SPIRIT. (DOC 58 kb) [file 13063_2016_1511_MOESM1_ESM.doc]

Figure 2. Schedule of enrolment, interventions, and assessments in line with SPIRIT

|  | **STUDY PERIOD** | | | | |
| --- | --- | --- | --- | --- | --- |
|  | **Enrolment** | **Allocation** | **Post-allocation** | | |
| **TIMEPOINT** | ***Baseline*** | **0** | ***3 months*** | ***6 months*** | ***12 months*** |
| **ENROLMENT:** |  |  |  |  |  |
| **Eligibility screen** | X |  |  |  |  |
| **Informed consent** | X |  |  |  |  |
| **Allocation** |  | X |  |  |  |
| **INTERVENTIONS:** |  |  |  |  |  |
| ***Blended treatment*** |  |  |  |  |  |
| ***Treatment-as-usual**** |  |  |  |  |  |
| **ASSESSMENTS:** |  |  |  |  |  |
| ***Demograhic variables*** | X |  |  |  |  |
| ***Current***  ***treatment*** | X |  |  |  |  |
| ***Diagnostic interview (M.I.N.I)*** | X |  |  |  | X |
| ***Depressive symptoms***  ***(PHQ-9)*** | X |  | X | X | X |
| ***Depressive symptoms***  ***(QIDS-SR16)*** | X |  | X | X | X |
| ***Quality of life***  ***(EQ-5D-5L)*** | X |  | X | X | X |
| ***Societal costs***  ***(TIC-P)*** | X |  | X | X | X |
| ***Treatment***  ***preference*** | X |  |  |  |  |
| ***Patient***  ***Expectancy (CEQ)*** | X |  |  |  |  |
| ***Working alliance (WAI-SF)***** |  |  | X |  |  |
| ***Technology alliance (TAI-SF)*** |  |  | X |  |  |
| ***Client satisfaction (CSQ)*** |  |  | X |  |  |
| ***Satisfaction with the online programme (SUS)***** |  |  | X |  |  |

*Treatment-as-usual is defined as the routine care that participants receive and is as such not time limited.

** Questionnaires taken from patients and therapists
